# Automated Heart Rate Detection in Seismocardiograms Using Electrocardiogram-Based Algorithms—A Feasibility Study

**DOI:** 10.3390/bioengineering11060596

**Published:** 2024-06-11

**Authors:** Evgenii Pustozerov, Ulf Kulau, Urs-Vito Albrecht

**Affiliations:** 1 Department of Digital Medicine, Medical Faculty OWL, Bielefeld University, 33615 Bielefeld, Germany; evgenii.pustozerov@uni-bielefeld.de; 2 Smart Sensors Group, Hamburg University of Technology (TUHH), 21073 Hamburg, Germany; ulf.kulau@tuhh.de

**Keywords:** seismocardiography, heart rhythm, accelerometers, electrocardiogram

## Abstract

In recent decades, much work has been implemented in heart rate (HR) analysis using electrocardiographic (ECG) signals. We propose that algorithms developed to calculate HR based on detected R-peaks using ECG can be applied to seismocardiographic (SCG) signals, as they utilize common knowledge regarding heart rhythm and its underlying physiology. We implemented the experimental framework with methods developed for ECG signal processing and peak detection to be applied and evaluated on SCGs. Furthermore, we assessed and chose the best from all combinations of 15 peak detection and 6 preprocessing methods from the literature on the CEBS dataset available on Physionet. We then collected experimental data in the lab experiment to measure the applicability of the best-selected technique to the real-world data; the abovementioned method showed high precision for signals recorded during sitting rest (HR difference between SCG and ECG: 0.12 ± 0.35 bpm) and a moderate precision for signals recorded with interfering physical activity—reading out a book loud (HR difference between SCG and ECG: 6.45 ± 3.01 bpm) when compared to the results derived from the state-of-the-art photoplethysmographic (PPG) methods described in the literature. The study shows that computationally simple preprocessing and peak detection techniques initially developed for ECG could be utilized as the basis for HR detection on SCG, although they can be further improved.

## 1. Introduction

Implementing accelerometer-based heart analysis techniques to analyze the cardiovascular system can make healthcare manageable in remote regions and regions where healthcare procedures, such as echocardiography, are unavailable. The accelerometers suitable for heart activity monitoring are common, low-cost, unobtrusive, and easy to use. One of the core methodologies to measure the activity of the cardiovascular system and the state of health in general is the analysis of the heart rhythm. The implementation of photoplethysmography (PPG) has made the study of heart rhythm affordable; at the same time, this method lacks precision in various conditions, is extremely sensitive to motion artifacts, and is known not to give reliable measurements for people with dark skin [1]. Recent studies show a common disparity between commercially available wearable technology heart rate (HR) monitors and methods based on electrocardiography (ECG) [2]; we might expect comparable precision from accelerometer-based methods.

Much research and many papers are dedicated to the ongoing research on reliable and robust R-peak detection algorithms for ECG. ECG signals are stable and are among the most reliable sources of information on heart rhythm. Nonetheless, the ECG has drawbacks: susceptibility to electromagnetic interference, the imperative for consistent electrode placement (prone to slipping or detachment), optimal skin-electrode impedance (which worsens with gel drying), and associated electrical hazards. Consequently, it may prove unsuitable for continuous, long-term monitoring, especially in daily life scenarios, and often entails discomfort. Portable alternatives, like the Holter monitor, have been utilized for continuous measurement, although they typically allow recordings of no more than 3 days [3]. Moreover, ECG demands the expertise of skilled clinical professionals. However, accessing a healthcare professional for this task may pose challenges, particularly in developing and rural locales.

Ballistocardiography (BCG) [4] and seismocardiography (SCG) [5] can be utilized instead of ECG when non-invasive monitoring of the mechanical aspects of cardiac function, such as body recoil movements or chest wall vibrations, is required. BCG is a non-invasive technique that measures the mechanical activity of the heart by detecting the body’s recoil movements as blood is ejected with each heartbeat. SCG, on the other hand, is a non-invasive method that records the vibrations of the chest wall induced by the heart’s mechanical actions, particularly the opening and closing of heart valves and the myocardial movements. The primary difference between the two lies in their focus: BCG measures the whole body’s recoil forces, while SCG captures localized chest wall vibrations.

BCG and SCG signals are more complicated to utilize as they are prone to disturbances from a wide range of internal and external noise sources. They are also dependent on the placement of the sensor and body composition. If we can precisely detect heart rhythm using BCG/SCG, we can perform heart rate variability (HRV) analysis without acquiring ECG signals, giving us much flexibility and many possible applications. For SCG, for instance, the well-detected heartbeats can be used to acquire the averaged SCG heartbeats to obtain information about the mechanical functioning of the heart derived from fiducial points.

The peak detection method can be presented in two steps:

1. Signal preprocessing (this mainly implies filters and basic mathematical operations), which has signal samples (points) on the input and the output;
2. Peak detection, which takes the signal samples and outputs the sequence of peak timestamps, a one-point pro heartbeat, which is then used to calculate HR and HRV parameters.

The recent review on the topic [6] depicts a wide variety of SCG applications, including HR detection. The paper presented by Garcia-Gonzales et al. [7] proposed using the continuous wavelet transform (CWT) on a filtered signal. Similar methods have also been previously used for ECG. The recent paper by Centracchio et al. [8] utilized template matching based on normalized cross-correlation, which showed promising results in patients with heart diseases. Another paper [9] used a template-based method to detect systoles and diastoles separately.

Other recent studies have acquired comparable precision using algorithms based on the Hilbert transform [10] and signal energy thresholding [11]. These are more accessible than deep learning techniques but are still computationally intensive. Both strategies are highly dependent on the cleanliness of the signal. Some papers propose machine-learning techniques to detect heartbeat intervals, such as convolutional neural networks (CNNs) for classification [12], U-Net neural networks for semantic segmentation [13,14], unsupervised segmentation [15], deep dominant frequency regressor [16], BiLSTM networks [17], and data-adaptive variational mode decomposition (VMD) [18]. Recent studies have also proved the possibility of recording SCG using cameras with a precision that exceeds the PPG method [19]. Those have shown promising results but are hard to implement on edge devices.

Sometimes, research only partially considers signals from various activities and new patients. Studies often test methods in isolation, limiting comparability across approaches. In addition, many research papers have included manual procedures, such as discarding signal parts affected by motion artifacts or other disturbances, which we still want to capture and process automatically. In a real-world scenario, making an algorithm work fully automated without interacting with the final user is necessary.

Although many papers are dedicated to developing peak detection algorithms for SCG signals, the knowledge and practice put into HR detection in ECG can land a basis for effective application in SCG. Morphologically, SCG heartbeat has similar features as ECG—mainly, the most prominent peak pro heartbeat (which in SCG case might be correspondent to either systole or diastole depending on sensor placement and selected axis) and similar frequency spectrum, which makes ECG peak detection and preprocessing methods applicable for SCG in the first precision.

We aim to compare various methods and techniques in a single framework based on data from multiple sources and to select the algorithm with the highest HR evaluation quality in SCG compared to ECG (the gold standard). For that, we identify a set of algorithms developed for ECG peak detection, apply them to the SCG signals derived from open-source datasets and new experimental data, and evaluate their accuracy. The detection quality for the best functioning algorithm is then compared with the SOTA JJ-interval detection algorithms developed directly for SCG in Section 4.

## 2. Materials and Methods

We proceed in three steps to clarify the research question:

1. In the first step, HR detection algorithms from ECG diagnostics were identified using a literature and software search.
2. In the second step, the precision of these algorithms was tested using a test data set of ECG (gold standard) and SCG data collected in parallel to identify the best methods. The quality of the method is determined by the highest precision in determining the HR (lowest deviation from the gold standard).
3. In the third step, the applicability and precision of the best algorithm are tested on real-life data in an experiment under resting conditions (sitting) and light activity (reading) interference.

### 2.1. Identification of ECG HR Algorithms and Implementation of Signal Preprocessing and Peak Detection Methods

#### 2.1.1. Identification

We investigated the recent systematic reviews [20,21,22] and code realizations for various techniques to select the set of peak detection and preprocessing methods for HR evaluation of ECG. At the beginning of the study, the choice was made to compare the algorithms already implemented in open-source code to have the possibility to assess and validate them. Utilizing the validated pipelines implemented in open-source packages helped us narrow the room for possible mistakes during code preparation and significantly diminish the implementation time. Therefore, non-open-source realizations of HR detection algorithms, such as those implemented in Kubious HRV software (v.4.0) [23], and methods presented in scientific papers without open-access code publication were excluded from the analysis.

We systematically searched available open-source packages for ECG processing and HRV analysis using the GitHub platform. We selected all relevant packages with at least 5 stars and which contained peak detection algorithms. We evaluated the algorithm implementations presented in the following packages: BioSPPy (v.2.2.2) [24], HeartPy (v.1.2.7) [25], HRV (v.0.2.10) [26], neurokit2 (v.0.2.7) [27], pyHRV (v.0.4.1) [28], PySiology (v.0.0.9.6) [29], RapidHRV (v.0.2.4) [30], and Systole (v.0.3.0) [31] packages. The neurokit2 package (v.0.2.7) [27] was selected as the most adequate for the research goals. It provides the most extensive set of well-described, widely accepted, and computationally non-greedy algorithms, which were most tested and underwent quality assessment.

The application of preprocessing and peak detection methods derived from a well-cited package allowed us to foster the transparency and reproducibility of our research, as we make it easy for researchers to cross-validate our study.

All calculations and visualization in the research were carried out using Python 3.11.4 programming language [32]. We developed the experimental framework based on the neurokit2 package (v.0.2.7) [27]. We tested the feasibility of the package’s peak detection methods and preprocessing pipelines, initially developed for ECG, on recorded SCG signals. We also provide open access to the source code developed during the preparation of this manuscript.

#### 2.1.2. Signal Preprocessing Pipelines

The following preprocessing pipelines were applied in the framework. Cleanup methods filter an ECG signal to remove noise and improve peak-detection accuracy. In total, 6 implemented cleanup methods were implemented and tested using the neurokit2 package together with our proposed filter approach and a raw signal bypass, Table 1.

Specified preprocessing methods were effectively utilized in practical applications as a stage in the heartbeat detection pipelines. Their aims are:

1. to eliminate the low-frequency component with no morphological features below the HR;
2. to eliminate high-frequency distortions induced by motion and other sources;
3. to eliminate the 50 Hz magnetically induced interference, interference currents in the body, and interference currents in the electrode leads;
4. in some cases, to eliminate substantial signal components not directly associated with the primary wave in the signal corresponding to the core fiducial point, usually R-peak (this applies to filters with a short bandpass zone).

Although 50 Hz filtering is irrelevant for HR detection in SCG signals, the presented filtering solutions contain low-pass and high-pass filters that are highly relevant to signals derived from accelerometers. In their work, Elgendi et al. [35] proved that cut-off frequencies influenced the quality of heartbeat detection and proposed the best cut-off frequencies for low-pass and high-pass filters. We anticipate the same will apply to SCG signals. The additional filtering method we present for comparison in the paper has shown the best artifact elimination with signal wave retention for further fiducial points analysis on previously obtained experimental data.

#### 2.1.3. Peak Detection Methods

Peak detection methods take the signal as an input and output the sequence of R-peaks (or corresponding core fiducial points on SCG). Altogether, 15 peak detection methods were compared in the testing framework—Table 2. The selected list includes well-cited and widely used algorithms in the literature suitable for real-time peak detection, starting from the classical Pan and Tompkins (1985) [33] algorithm and up to the introduced in the 2023 approach by Emrich et al. [37]. The methods were implemented using the neurokit2 package.

The chosen methods are based on standard signal processing techniques and are computationally inexpensive, which makes them feasible for real-time calculations. We do not target complex machine learning and, especially, deep learning techniques to make calculations on cheaper edge devices possible. This makes the final solution affordable and feasible for implementation on wearable devices, avoiding high computational complexity.

We also considered only the algorithms that work fully automatically, without any anticipated ad-hoc configuration from the user side.

### 2.2. Application of the Identified Algorithms to the Test Dataset

#### 2.2.1. Evaluation Strategy

We will refer to the SCG signal’s main anchor points corresponding to the R-peaks on the ECG as J-peaks. In most cases, this point is the peak of the SCG’s systolic or diastolic complex, depending on sensor placement and selected axis.

For each recording, we ran calculations on the 60 s episode, which gives enough signal length to make a precise HR measurement that is not strongly affected by the HRV and signal cut and is widely used in other papers [49]. HR was evaluated based on the number of detected peaks and the signal fragment’s length. To avoid a mismatch between the number of peaks resulting from a non-complete heartbeat at the beginning and the end of the fragment (e.g., when the last R-peak is included in the fragment while the last J-peak is not), we deleted the j-peaks before the first R-peak, the last R-peak, and all J-peaks after the last R-peak. Then, these sequences of detected peaks were used to calculate the HR. The HR was calculated as 60 s divided by the mean length of the RR interval for the segment.

We evaluated the detection accuracy of J-peaks on the SCG with the following approach:

- the detected R-peaks on ECG were taken as ground truth;
- the first peak on SCG that hit the particular RR interval was treated as a true positive (TP), while every following peak in the interval was treated as a false positive (FP);
- each RR interval without any peaks on SCG hitting it is treated as a false-negative (FN).

Precision (positive predictive value), recall (sensitivity), and F1-score were calculated using the standard formulas and TP, FP, and FN values:

(1) $$\mathrm{Precision}=\frac{\mathrm{TP}}{\mathrm{TP}+\mathrm{FP}},$$

(2) $$\mathrm{Recall}=\frac{\mathrm{TP}}{\mathrm{TP}+\mathrm{FN}},$$

(3) $$\mathrm{F1}=\frac{2\cdot \mathrm{Precision}\cdot \mathrm{Recall}}{\mathrm{Precision}+\mathrm{Recall}}.$$

We did not specify the particular window size after the R-peak for the J-peak to hit to be treated as TP, as it does not affect the accuracy of HR calculation (considering it correctly hit the RR interval).

#### 2.2.2. Reference Dataset

We used the CEBS dataset [7] available at PhysioNet to compare the methods and choose the best approach. The SCG recording in this dataset was acquired using a triaxial accelerometer (LlS344ALH, ST Microelectronics) and a filter with a bandwidth between 0.5 Hz and 100 Hz. The conventional ECG (leads I and II, respectively) was recorded with a bandwidth between 0.05 Hz and 150 Hz. The subjects were asked to be awake and supine on a comfortable conventional single bed during the measurement. The dataset consists of records obtained from 20 subjects.

The dataset contains recordings from a single accelerometer patch with combined axes for 20 healthy subjects in three stages: before listening to the music (up to 5 min of recordings), during music listening (up to 45 min), and after the music (up to 5 min). We evaluated algorithms based on the signals recorded during the second stage, which contains data for the most stable part of the experiment.

Before applying the algorithms, we downsampled the dataset signals to 1 KHz sampling frequency. We used the I lead from ECG to detect the R-peaks.

The complete description of the dataset is available in the original paper [7].

### 2.3. Testing the Best Algorithm in a Real-Life Setting in Rest and Interfering Activity Conditions

#### 2.3.1. Experiment

We conducted an experiment to evaluate the selected best method on new test data. SCG and ECG were measured in subjects without heart diseases in two modes:

- 5 min seated, physical rest;
- 5 min seated, reading the book aloud without any additional physical activity.

The reading was selected as an exercise as speaking is currently among the most critical and frequent sources of disturbances in the SCG. The interference from muscle movements and rapid inhalations during speaking greatly disturbs the waves related to the circulatory system in SCGs.

The inclusion criteria were the capacity to give consent, 18–60 years of age, any gender, fit for duty, and no heart or cardiovascular disease. The exclusion criteria were lack of capacity to consent and known allergy/intolerance to electrode gel/wound dressing.

**Figure 1:**
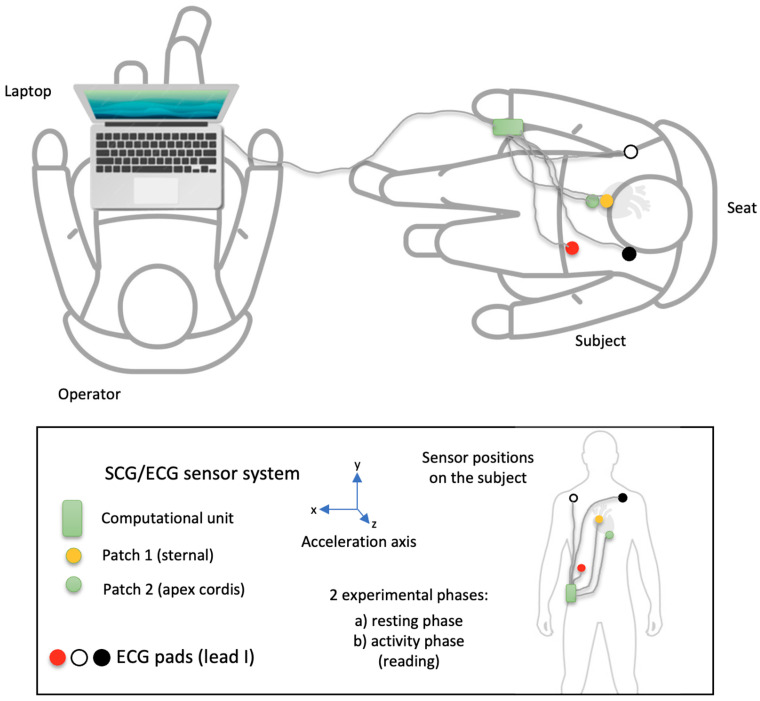
The experimental scheme of the measurement. Patch 1 (yellow) is for sternal accelerometers; patch 2 (green) is for apex cordis accelerometers. The other patches belong to the reference ECGs.

Study procedure: The study participants were informed about the study. The inclusion and exclusion criteria were requested and checked when they consent to participate in the study. Age, weight, height, and body mass index (BMI) were collected and recorded. While seated, the study participants were taped with 2 SCG sensors and ECG electrodes (Figure 1). The participants were asked to relax and avoid physical activity during the first part of the measurement. The participants were asked to read out loud from a book while avoiding other types of physical activity during the second part of the measurement. The duration of each part of the experiment was 5 min.

Schedule: The subject is fitted with the accelerometer sensors (sternal, apex cordis) and the 1-channel ECG. Before taking the measurements, the operator and the test subject check the available equipment and its functionality. The positions of the reference sensors are approved. The operator then tests the connectivity and checks the data recording. When all readings are satisfactory and no adjustments are required, the operator starts logging data, and the measurement begins. After the last experiment, the logging is stopped, the data is stored and backed up, and the operator and the test subject are released from their positions. The sensors and the ECG are then removed.

A fused sensor system of commercially available 3D accelerometers was used to determine the micro-accelerations. The ECG (electrical potential differences) serves as a reference system.

The study protocol was approved by the Westphalia-Lippe Ethics Committee (act ref. l 2024-134-f-S, 19 March 2024).

#### 2.3.2. Sensor System

**Figure 2:**
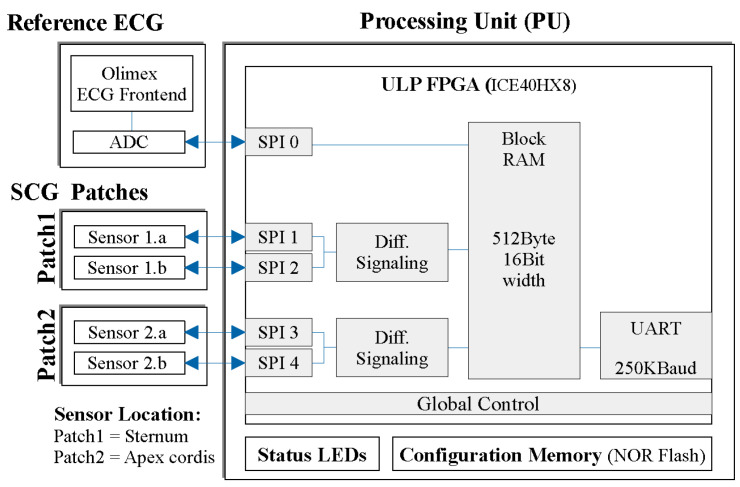
A block diagram of the measurement system, showing the primary hardware modules (such as sensor patches, ECG, and processing unit), along with the main firmware blocks within the preprocessing unit (PU).

The two sensor patches and the ECG are connected to a processing unit (PU) to interface, preprocess, and transmit the measured data. Custom implementation was necessary to support differential Seismocardiogram (SCG) sensing for each patch and ensure time-synchronous acquisition of ECG data, allowing for clock-synchronous interfaces (see Figure 2). The system is described in detail in [50,51].

## 3. Results

In Section 3, we describe first the algorithm selection approach and then the results of testing the selected algorithm on the experimental data.

### 3.1. Finding the Best Approach Using the CEBS Dataset

For the comparison between algorithms, we took five randomly selected 60-s episodes of the music-listening part of the recording. We applied each preprocessing method (8) and peak detection method (15) for five 60-s parts of 20 recordings, totaling 12,000 runs.

One of the 15 algorithms, emrich2023, could not work with the SCG (it outputted empty results). All the other combinations provided results with various detection qualities.

The number of heartbeats was calculated for each of the selected sixty-second recordings. The best method was chosen from the iteration with the lowest HR difference between HR detected using ECG and SCG signals. The correctness of the detected R-peaks on the ECG (ground truth) was additionally manually checked using signal visualization to ensure proper labeling. Table 3 shows the resulting accuracy metrics for the top three detection methods, the complete table with all methods is presented in Supplementary Materials Table S1.

**Figure 3:**
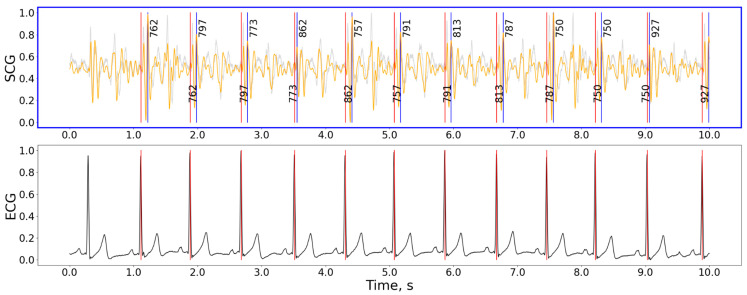
The detection example for the hamilton2002 preprocessing and nabian2018 peak detection combination of methods. Gray curve—raw signal, orange curve—signal after being processed with the hamilton2002 pipeline, black curve in the bottom plot—ECG signal, red vertical lines—timestamps of R-peaks detected on ECG, blue vertical lines—J-peaks detected on SCG, the numbers next to blue vertical lines show the distance from the previous and to the next peak in ms. For the selected interval: precision = 100%, recall = 100%, F1-score = 1.00, HR: 61.9 bpm, *n*(j_peaks) = *n*(R_peaks) = 9. The accuracy calculation does not include peaks close to the plot’s border. Signal magnitudes are normalized for demonstrational purposes.

The best peak detection methods were the nabian2018, neurokit, and elgendi2010. Figure 3 shows an example of the algorithm working on a 10-s signal fragment.

The combination of the nabian2018 peak detection method with a hamilton2002 has shown the best quality of peak detection, resulting in an average error in HR estimation below 1 bpm. nabian2018 also has a very low computation complexity. This algorithm employs a sliding window of 400 ms duration, moving one sample at a time to scan the entire signal. During each scan, the highest point within the window is identified as a potential R peak when it occurs in the center of the window. This process allows for the detection of resembling R peaks across the entirety of the input ECG signal. The nabian2018 method has shown good results partly because it targeted the highest point in the windows and not the first high point in the window, unlike many other HR detection algorithms based on peak detection principles. The Neurokit2 package [27] contains a simplified version of the Nabian et al. algorithm compared to the one presented in the original paper [44], omitting the post-detection with additional restrictions on peak amplitudes and interpolating missing peaks. We implemented both approaches in the code and compared them back-to-back. The resulting precision for HR detection for the version without peaks post-processing has shown better results for SCG signals.

Table 4 compares various preprocessing techniques when combined with the nabian2018 peak detection method. The cleanup methods had a relatively minor effect on the result.

### 3.2. Testing the Approach on the Experimental Data

The previous section shows that the HR evaluation method initially developed for ECG can be successfully applied to SCG signals for subjects lying still and in laboratory conditions. To evaluate the application of the selected best algorithm to other conditions and recording hardware, we ran experiments with subjects performing varying activities. The measurements were taken from 3 healthy subjects (male, age = 42.7 ± 6.9) in accordance with the protocol described in Section 2.

We applied the chosen best preprocessing and peak detection methods combination (1) to each patch (2) and axes (3) for five 1-min signal parts per each recording (6), totaling 180 runs. The results for each subject and averaged values for resting and activity interference recordings are shown in Table 5.

**Figure 4:**
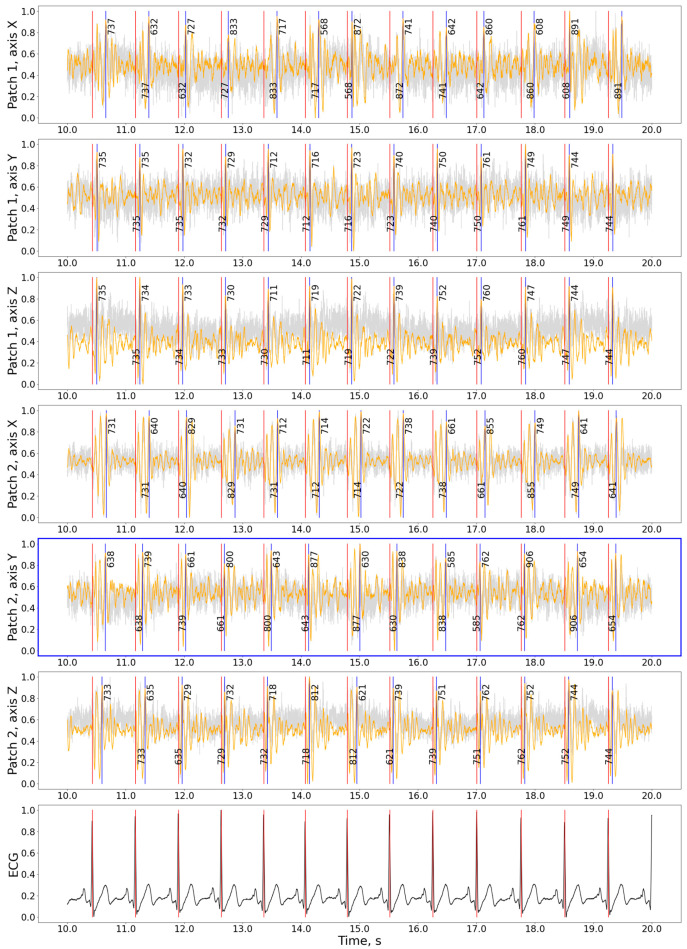
The detection results for the hamilton2002 preprocessing and nabian2018 peak detection combination on the experimental data. Gray curve—raw signal, orange curve—signal after being processed with hamilton2002 algorithm, black curve in the bottom plot—ECG signal, red vertical lines—timestamps of R-peaks detected on ECG, blue vertical lines—J-peaks detected on SCG, the numbers around blue vertical lines show the distance from the previous and to the next peak in ms. The patch with the best precision is shown with a blue frame. For the selected interval: precision = 100%, recall = 100%, F1-score = 1.00, HR: 81.2 bpm, *n*(j_peaks) = *n*(R_peaks) = 12. The accuracy calculation does not include peaks close to the plot’s border. Signal magnitudes are normalized for demonstration purposes.

Contrary to the CEBS dataset with a single integrated SCG signal, our data allows for selecting patches and axes before HR evaluation. The signal examples for various patches and axes are shown in Figure 4. Similar examples for other subjects and states are presented in Supplementary Materials, Figures S1–S6. The patches for each measurement were selected based on the minimal difference between HR measured in SCG and ECG. In some cases, the difference in precision between various patches was minimal.

There was no statistical difference between HR measured with ECG and SCG for signals recorded while resting (paired two-sided t-test, *n* = 5; t: 0.365 (*p* = 0.733), −2.448 (*p* = 0.071), 1.102 (*p* = 0.332); normal distribution according to the Kolmogorov–Smirnov test; KS: 0.500 (*p* = 0.111), 0.500 (*p* = 0.112), 0.501 (*p* = 0.111) for HR diff distributions for subjects 1, 2 and 3 respectively). For the recordings with interference, the HR measured from SCG signals tends to be lower than that measured from ECG. The average absolute difference between the recordings compared for all activities was 3.3 ± 3.8 bpm, slightly less than reported for commercially available HR monitors [2].

As for the computational complication, the initial realization of the Nabian et al. algorithm in the neurokit2 package was already among the fastest implemented algorithms with a processing time comparable with the Pan–Tomkins algorithm. The further optimization of the algorithm (compare-and-swap utilization, inner loop elimination by comparing with a previous peak) and code implementation in the Rust programming language [52] allowed us to reduce the processing time for a 5-min recording from 139.38 ms to 0.20 ms when executed on a MacPro M1 2020 16 GB machine, which leaves a sufficient resource margin to be applicable for runtime execution on practically any edge device.

### 3.3. Analysis of Heartbeat Detection Precision

The detection of HR on the 60-s interval allows for precise HR evaluation even when particular peak occurrence times were not indicated correctly. At the same time, detecting precise beat-to-beat intervals enables HRV analysis and instant HR evaluation, although being especially sensitive to disturbances that affect precise peak time occurrence. We calculated the precision of heartbeat interval evaluation between RR and JJ pairs. For the CEBS dataset, the mean difference between the RR and JJ intervals detected with the selected method was 1.8 ± 1.7 ms (with the mean RR-interval: 849.7 ± 122.1 and the mean J-interval: 849.5 ± 125.1; 5-min recording per subject, 20 subjects). The same values on the new experimental data were 20.8 ± 32.0 ms and 129.8 ± 90.2 ms for relaxation and recorded during interference accordingly (with the mean R-interval: 847.9 ± 122.3 and fg; the mean J-interval: 847.7 ± 125.4 and fg; 5-min recording per subject, 3 subjects each). The other investigated methods have shown lower precision in comparison to the nabian2018 and hamilton2002 combination.

**Figure 5:**
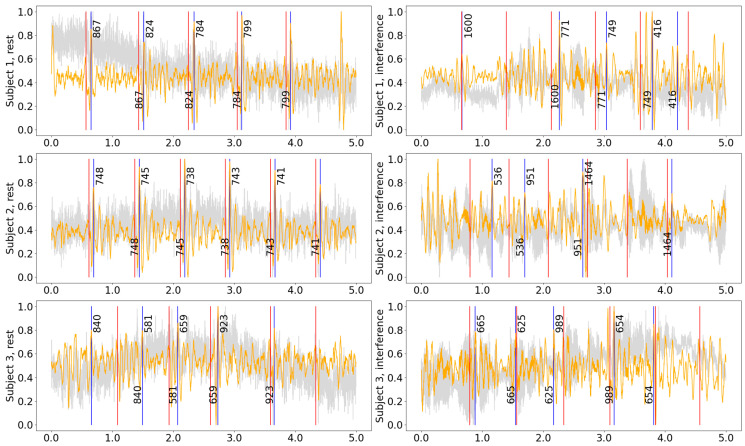
Five-second samples from the middle of the recording for different subjects while resting and during reading interference. All measurements were acquired from Patch 1, axis Z. Gray curve—raw signal, orange curve—signal after being processed with hamilton2002 pipeline, red vertical lines—timestamps of the R-peaks detected on the ECG, blue vertical lines—the J-peaks detected on the SCG, the numbers next to blue vertical lines show the distance from the previous and to the next peak in ms.

Figure 5 compares the signals recorded during rest and while speaking for three subjects in the experiment. Although the HR can be evaluated for a recording made during reading inference using approximations, analysis of HRV for those signals could not be performed reliably as speech strongly affects the time when the prominent peaks appear in the signal. For that purpose, the intelligent system might be used to distinguish between intervals when only HR and where both HR and HRV characteristics could be evaluated with precision within a defined range of values.

**Figure 6:**
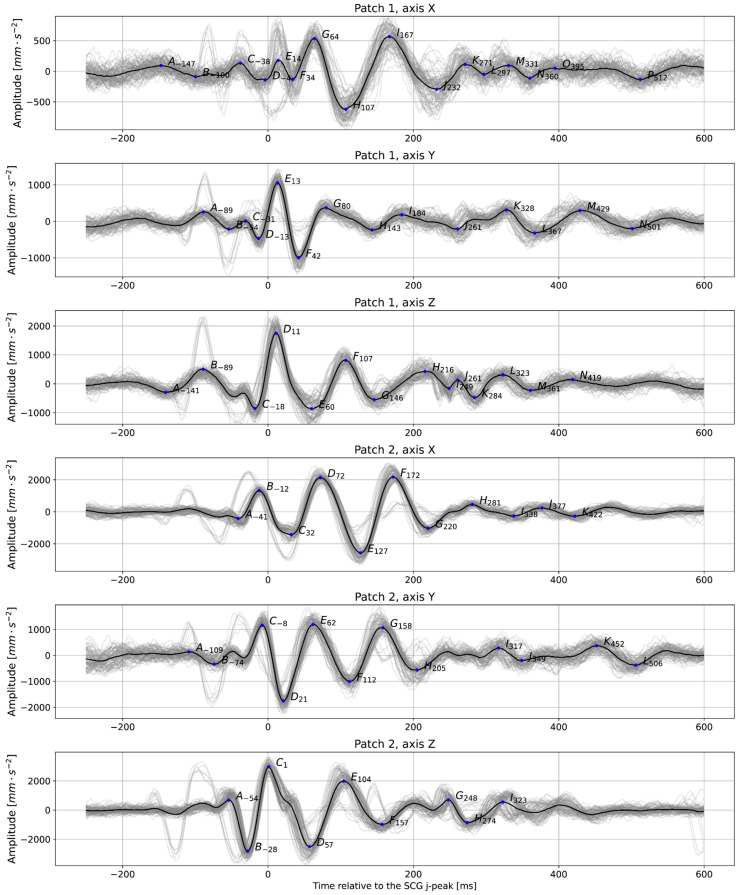
Averaged plots were created based on detected anchor points (in this case, J-peaks detected on the signal from Patch 1, axis Z were taken as anchor points for all signals); subject 2, relaxed, t = 60 s. The prominent peaks are labeled with letters. The number next to the letter shows the distance between each peak and the anchor point. Gray lines show the individual trajectories of each heartbeat.

The precise detection of heartbeats on the SCG is also essential, as those serve as anchor points for building averaged heartbeat images, which can be used for fiducial points analysis. Figure 6 shows an example of an averaged heartbeat in various axes derived from our data. These plots can evaluate various mechanical characteristics of heart activity, such as waves corresponding to valves closing and opening.

## 4. Discussion

The selected combination of peak detection and preprocessing methods showed good results while being computationally very unburdened. The current study proves that a relatively simple algorithm can be used to evaluate HR on SCG, and it would be the same algorithm that works for ECG, which makes the procedure methodologically straightforward. Table 6 compares the current work with the previous results from the literature. In general, we can see commensurate results for all methods. For comparability reasons, we only present here the results from papers that tested algorithms on the CEBS [7] dataset that serves as a common benchmark. Detecting HR using SCGs in conditions such as in the CEBS dataset with agreeable accuracy was proven to be a feasible task for many algorithms. The most prominent algorithm can be chosen as the one with the lowest resource consumption, fastest runtime, and most uncomplicated calculation procedure. Here, the algorithm by Nabian et al. [44] is favorable due to its simplicity and proof of its reliable work for another type of biosignal—ECG.

More research should target SCGs during activities and interferences. For the clean recording with no activity, algorithms as simple as the one we have selected in this research proved to show good precision. At the same time, the experiment showed only moderate precision while speaking interference took place. The algorithms should be further adapted to work in various conditions, such as during disturbances and physical activities.

Another critical question is the choice of the best patch and axis to evaluate heart rhythm. Several approaches can be proposed: ad hoc (choosing the patch and axis before evaluating the HR) and post hoc (detecting HR in all patches and axes and selecting the most reliable evaluation). In our paper, we have selected the patch and axis corresponding to the smallest difference between HR in ECG and SCG. In a real-world scenario where ECG is not available, the possible solution could be using the JJ sequence with the lowest heart rate variability characteristic (such as SDNN—standard deviation of normal JJ-intervals), the power density of the signal spectrum in the interval, or another similar characteristic.

It might sound reasonable that the Z-axis might give the best result, but this is only sometimes true. In some cases in our data, the X or Y axes gave the best result. Also, systolic and diastolic peaks are variously prominent in different axes and locations of the chest surface; this can be clearly seen in Figure 4. The study [10] uses a power spectral density (PSD) plot to identify the frequency domain’s most informative axis per sensor. We used just the HR_diff metric. It might also be reasonable to use SDNN.

HR is more accessible to detect than HRV, which requires precise evaluation of beat-to-beat intervals and not only the right amount of them. HRV is especially difficult to calculate because the peaks on SCG are highly prone to movement artifacts, vary more, and are not as stable in form as the R peaks on the ECG signal. Other problems include breathing, especially at the J-peaks, affecting the exact time when the R-peak and J-peak occur, and the inter-subject variability in SCG morphology (which could be possibly dealt with employing methods not relying much on apriori assumption about the signal shape or specific fiducial points to recognize the heartbeat).

The results obtained from evaluating the precise lengths of heartbeat methods show that the selected method is not reliable enough to detect HRV under interference and also has a large margin for improvement for signals recorded during relaxation. This also raises a question of the applicability of test methods that have shown good results using CEBS data only to new data acquired in less strict conditions than in the respective experiment.

## 5. Conclusions

Our study demonstrates that relatively simple processing and peak detection methods initially developed for ECG can serve as a foundation for HR detection in SCG data, albeit with room for further refinement, especially for recording made during physical activity. Combining Nabian et al.’s detection method with a proper preprocessing pipeline can effectively measure HR in various subjects. In ideal conditions, J peaks are stable enough to perform HRV analysis. Detected J-peaks allow us to obtain average heartbeat images on SCG and analyze fiducial points on SCG to characterize heart-vessel system conditions.

The described methods could be applied in real-life scenarios with a methodology that classifies the level of physical activity, external disturbances, and signal quality. Implementing such systems will empower a computationally simple yet reliable method and devices for remote monitoring, especially in countries where healthcare accessibility is limited.

## Figures and Tables

**Table 1:** Description of signal preprocessing pipelines.

Codename	Source	Description
neurokit	Neurokit2 package [27]	0.5 Hz high-pass Butterworth filter (order = 5), followed by powerline filtering (50 Hz)
biosppy	Biosppy package [24]	a finite impulse response filter with the order defined as [0.3 × sampling rate] with bandpass cut-off frequencies 3 and 45 Hz
pantompkins1985	Pan and Tompkins (1985) [33]	a 1-order bandpass Butterworth filter with 5 and 15 Hz cut-off frequencies
hamilton2002	Hamilton et al. (2002) [34]	a combination of 1-order Butterworth 8 Hz high-pass and 16 Hz low-pass filters
elgendi2010	Elgendi et al. (2010) [35]	a 2-order bandpass Butterworth filter with 8 and 20 Hz cut-off frequencies
current_paper	Current paper	a 4-order bandpass Butterworth filter with 5 and 35 Hz cut-off frequencies
engzeemod2012	Lourenco et al. (2012) [36]	a 5-order bandstop Butterworth filter with 48 and 52 Hz cut-off frequencies

**Table 2:** Description of peak detection methods.

Codename	Source	Description
neurokit	Neurokit2 package [27]	QRS complexes are detected based on the steepness of the absolute gradient of the ECG signal; subsequently, R-peaks are detected as local maxima in the QRS complexes
pantompkins1985	Pan and Tompkins (1985) [33]	an algorithm based on dynamically changing thresholds
hamilton2002	Hamilton (2002) [34]	adaptive thresholding
zong2003	Zong et al. (2003) [38]	a low-pass filter, slope sum function, and a decision rule
martinez2004	Martinez et al. (2004) [39]	combined adaptive filters
christov2004	Christov et al. (2004) [40]	two parallel running algorithms with a combination of three adaptive thresholds: steep-slope, integrating threshold for high-frequency signal components, and beat expectation threshold
gamboa2008	Gamboa et al. (2008) [41]	the first derivative and restrictions on possible RR lengths
elgendi2010	Elgendi et al. (2010) [35]	potential blocks generated based on two moving averages and the following thresholding
engzeemod2012	Lourenco et al. (2012) [36]	5-s intervals to determine adaptive threshold linearly changing in defined intervals
manikandan2012	Manikandan and Soman (2012) [42]	Shannon energy envelope (SEE)
kalidas2017	Kalidas and Tamil (2017) [43]	stationary wavelet transform (SWT)
nabian2018	Nabian et al. (2018) [44]	Pan-Tompkins inspired algorithm with moving windows and highest peak detection
rodrigues2021	Sadhukhan and Mitra (2012) [45], Gutiérrez-Rivas et al. (2015) [46], and Rodrigues et al. (2021) [47]	double derivative, squaring, moving window integration as preprocessing and a finite-state-machine for decision-making
emrich2023	Koka et al. (2022) [48] and Emrich et al. (2023) [37]	the fast natural visibility graph (FastNVG) algorithm based on the visibility graph detector; the algorithm transforms the ECG into a graph representation and extracts exact R-peak positions using graph metrics
promac	Neurokit2 package [27]	combination of several R-peak detectors in a probabilistic way: for a given peak detector, the binary signal representing the peak locations is convolved with a Gaussian distribution, resulting in a probabilistic representation of each peak location; the procedure is repeated for all selected methods, accumulating the resulting signals; a threshold is used to accept or reject the peak locations

**Table 3:** Comparison of peak detection algorithms in combination with the best passing preprocessing pipeline.

Rank	Detection Method	Preprocessing Method	HR (SCG), bpm	HR (ECG), bpm	HR diff, bpm	Precision	Recall	F1-Score
1	nabian2018	hamilton2002	70.8 ± 9.8	70.1 ± 9.8	0.9 ± 2.4	98.7 ± 3.1	99.7 ± 0.9	0.992 ± 0.018
2	neurokit	hamilton2002	85.0 ± 21.5	70.1 ± 9.8	15.0 ± 21.0	85.6 ± 17.0	99.4 ± 1.5	0.91 ± 0.11
3	elgendi2010	hamilton2002	86.0 ± 22.0	70.2 ± 9.8	15.8 ± 20.2	84.3 ± 16.7	98.9 ± 2.1	0.901 ± 0.106

**Table 4:** Comparison of preprocessing pipelines in combination with the peak detection method from Nabian et al. [44].

Rank	Detection Method	Preprocessing Method	HR (SCG), bpm	HR (ECG), bpm	HR diff, bpm	Precision	Recall	F1-Score
1	nabian2018	hamilton2002	70.8 ± 9.8	70.1 ± 9.8	0.9 ± 2.4	98.7 ± 3.1	99.7 ± 0.9	0.992 ± 0.018
2	nabian2018	pustozerov2024	69.9 ± 9.5	70.1 ± 9.8	0.9 ± 2.1	98.6 ± 4.5	98.3 ± 4.5	0.985 ± 0.043
3	nabian2018	elgendi2010	71.3 ± 10.1	70.1 ± 9.8	1.4 ± 3.6	92.0 ± 9.7	93.4 ± 9.4	0.926 ± 0.093
4	nabian2018	pantompkins1985	71.2 ± 10.0	70.1 ± 9.8	1.5 ± 3.9	98.0 ± 4.7	99.4 ± 1.8	0.986 ± 0.029
5	nabian2018	biosppy	70.3 ± 9.5	70.8 ± 9.4	1.8 ± 3.9	98.3 ± 4.5	97.7 ± 5.6	0.979 ± 0.045
6	nabian2018	none	70.3 ± 9.5	71.5 ± 9.9	2.5 ± 6.7	98.0 ± 4.8	96.7 ± 8.4	0.972 ± 0.061
7	nabian2018	engzeemod2012	70.3 ± 9.5	71.7 ± 10.0	2.7 ± 7.2	98.1 ± 4.8	96.6 ± 8.8	0.971 ± 0.063
8	nabian2018	neurokit	71.1 ± 9.6	71.4 ± 9.8	3.2 ± 6.8	97.0 ± 5.3	96.8 ± 8.3	0.967 ± 0.060

**Table 5:** The resulting precision of selected methods when applied to the experimental data (*n* = 30), detection method: nabian2018, preprocessing method: hamilton2002.

Subject	State	Best Patch and Axes	HR (SCG), bpm	HR (ECG), bpm	HR diff, bpm	*p*-Value	Precision	Recall	F1-Score
1	Rest	Patch1_z	67.4 ± 0.7	67.4 ± 0.6	0.1 ± 0.1	0.733	100.0 ± 0.0	100.0 ± 0.0	1.0 ± 0.0
1	Interference	Patch1_y	72.4 ± 1.8	76.6 ± 1.1	4.2 ± 1.1	0.001	89.2 ± 3.9	83.9 ± 3.7	0.864 ± 0.037
2	Rest	Patch0_z	83.5 ± 2.7	83.5 ± 2.7	0.0 ± 0.0	0.071	100.0 ± 0.0	100.0 ± 0.0	1.0 ± 0.0
2	Interference	Patch1_z	82.2 ± 2.9	91.8 ± 1.3	9.7 ± 2.5	0.001	98.0 ± 1.2	87.7 ± 3.6	0.926 ± 0.025
3	Rest	Patch1_x	68.6 ± 2.1	68.3 ± 1.6	0.3 ± 0.6	0.332	99.7 ± 0.6	100.0 ± 0.0	0.999 ± 0.003
3	Interference	Patch1_x	73.6 ± 1.1	79.1 ± 1.4	5.5 ± 1.9	0.003	92.5 ± 2.3	86.2 ± 2.0	0.892 ± 0.018
All	Rest	various	73.2 ± 7.8	73.1 ± 7.8	0.1 ± 0.3	0.278	99.9 ± 0.4	100.0 ± 0.0	1.000 ± 0.002
All	Interference	various	76.1 ± 4.9	82.5 ± 7.0	6.5 ± 3.0	<0.001	93.2 ± 4.5	86.0 ± 3.4	0.894 ± 0.036
All	Both	various	74.6 ± 6.6	77.8 ± 8.7	3.3 ± 3.8	<0.001	96.6 ± 4.6	93.0 ± 7.5	0.947 ± 0.059

**Table 6:** Comparison with the previous work.

N	Paper	Method	Metric and Value
1	Current paper	moving window and simple peak detection	sensitivity, 99.7 ± 0.9%; precision, 98.7 ± 3.1%; F1-score, 0.992 ± 0.018; HR diff, bpm = 0.9 ± 2.4
2	Prithvi et al. [12]	convolutional neural network (CNN)	sensitivity, 98%; precision, 98%
3	Mora et al. [15]	unsupervised segmentation	sensitivity, 98.5 ± 1.2%; precision, 98.6 ± 1.2%; specificity, 98.6 ± 1.2%
4	Duraj et al. [14]	U-Net-based semantic segmentation	sensitivity, 99.9%; precision, 97%
5	Chen et al. [17]	BiLSTM network	sensitivity, 97%; precision, 98%
6	Choudhary et al. [18]	data-adaptive variational mode decomposition (VMD)	sensitivity, 97.4%; precision, 97.4%; accuracy, 95.1%

## Data Availability

The anonymized datasets generated during the study are available from the corresponding author upon reasonable request.

## Supplementary Materials

The following supporting information can be downloaded at: https://www.mdpi.com/article/10.3390/bioengineering11060596/s1, Table S1: Comparison of peak detection algorithms in combination with the best passing preprocessing pipeline (all methods); Figures S1–S6: The detection results for the hamilton2002 preprocessing and nabian2018 peak detection combination on the experimental data (subjects 1–3, relaxed and with speaking interference).

## Computer Code Availability Statement

The codebase for all calculations in the paper is available under the following link: https://gitlab.ub.uni-bielefeld.de/ag4-digitale-medizin/papers/bioengineering-11-00596.
