# Supplementary material for: Automated Heart Rate Detection in Seismocardiograms Using Electrocardiogram-Based Algorithms—A Feasibility Study
Source: Bioengineering (Basel). 2024 Jun 11;11(6):596. doi: 10.3390/bioengineering11060596 (PMC11200605; doi:10.3390/bioengineering11060596)
Supplement: Supplementary file 1 [file bioengineering-11-00596-s001.zip › bioengineering-3015063-supplementary.pdf]

bioengineering

MDPI

Supplementary material

Table S1. Comparison of peak detection algorithms in combination with the best passing preprocessing pipeline.

Rank	Detection method	Preprocessing method	HR (SCG), bpm	HR (ECG), bpm	HR diff, bpm	Precision	Recall	F1-score
1	nabian2018	hamilton2002	70.8±9.8	70.1±9.8	0.9±2.4	98.7±3.1	99.7±0.9	0.992±0.018
2	neurokit	hamilton2002	85.0±21.5	70.1±9.8	15.0±21.0	85.6±17.0	99.4±1.5	0.91±0.11
3	elgendi2010	hamilton2002	86.0±22.0	70.2±9.8	15.8±20.2	84.3±16.7	98.9±2.1	0.901±0.106
4	promac	elgendi2010	86.6±18.6	70.2±9.8	16.6±17.5	81.8±14.7	98.2±4.8	0.885±0.098
5	manikandan2012	elgendi2010	89.5±21.5	70.1±9.4	19.4±19.7	81.0±15.8	99.3±1.2	0.884±0.104
6	engzeemod2012	engzeemod2012	68.4±25.2	68.4±15.3	19.7±21.8	85.5±21.0	79.2±26.9	0.765±0.243
7	pantompkins1985	elgendi2010	100.2±28.0	70.3±9.7	30.0±28.1	68.7±16.0	92.8±7.9	0.775±0.101
8	kalidas2017	elgendi2010	100.5±24.0	70.1±9.8	30.5±24.5	72.8±17.8	99.4±1.8	0.828±0.12
9	zong2003*	neurokit	117.0±20.3	90.8±27.9	33.0±22.7	73.7±18.2	95.2±8.1	0.813±0.104
10	hamilton2002	neurokit	108.9±24.9	72.4±10.2	36.6±24.6	65.9±14.7	95.2±6.1	0.767±0.096
11	rodrigues2021	biosppy	113.3±18.0	71.4±9.0	42.0±20.0	63.7±13.6	98.6±3.8	0.767±0.098
12	christov2004*	none	149.1±80.7	135.8±91.0	54.9±57.4	79.3±24.9	88.0±17.1	0.788±0.177
13	gamboa2008	neurokit	127.8±25.9	70.6±10.6	57.2±20.3	56.2±11.0	99.1±3.5	0.711±0.076
14	martinez2004*	elgendi2010	187.2±49.6	125.6±35.5	71.0±53.9	55.1±22.6	79.3±20.1	0.619±0.182
15	emrich**	none	-	-	-	-	-	-

* These methods inadequately measured HR on ECG and were excluded from the comparison; ** This method did not function and could not provide any output.

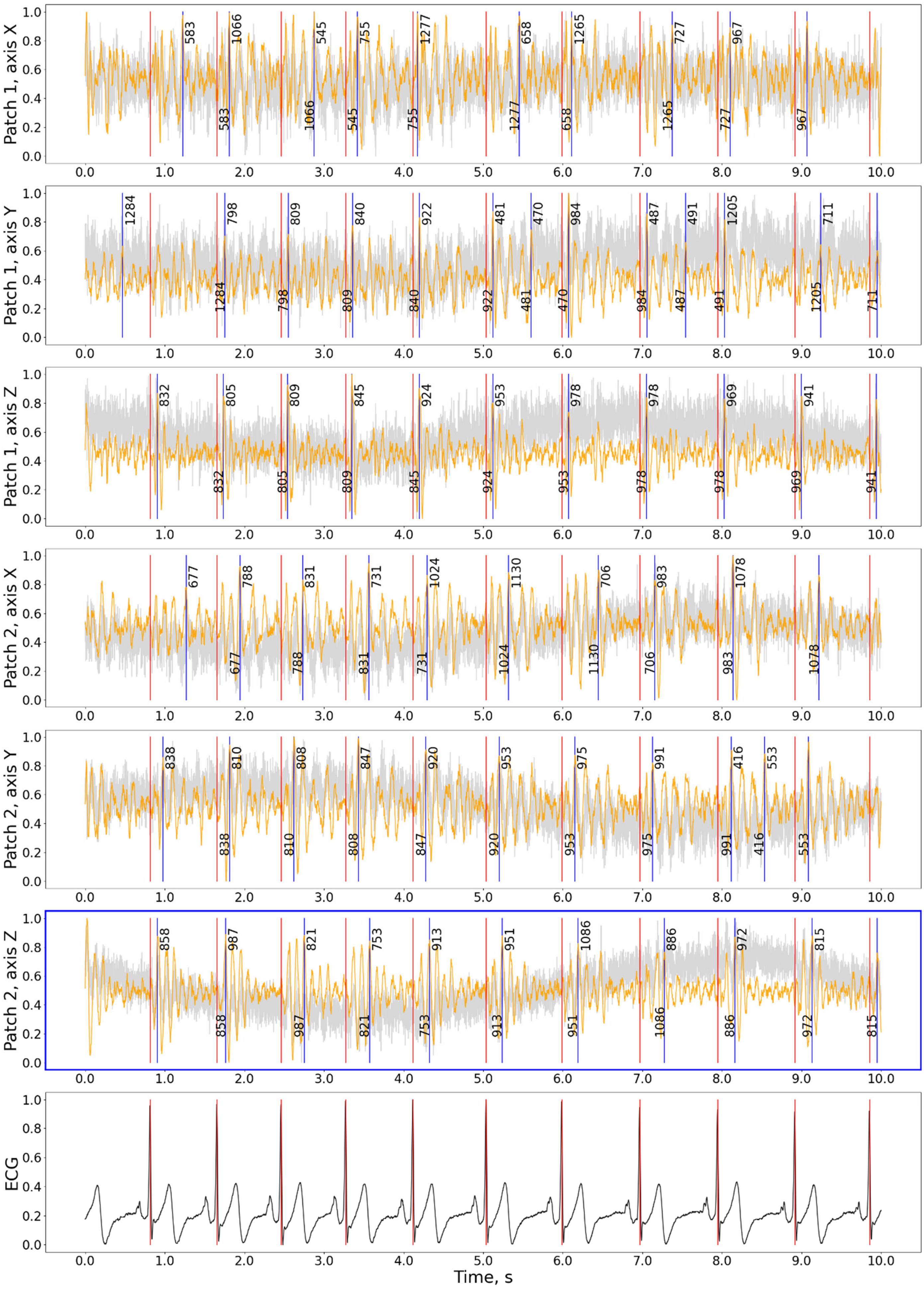
Patch 1, axis X
Patch 1, axis Y
Patch 1, axis Z
Patch 2, axis X
Patch 2, axis Y
Patch 2, axis Z
ECG
Time, s

Figure S1. The detection results for the hamilton2002 preprocessing and nabian2018 peak detection combination on the experimental data. Subject 1, relaxed. Gray curve – raw signal, orange curve – signal after being processed with hamilton2002 algorithm, black curve in the bottom plot – ECG signal, red vertical lines – timestamps of R-peaks detected on ECG, blue vertical lines – J-peaks detected on SCG, the numbers around blue vertical lines show the distance from the previous and to the next peak in ms. The patch with the best precision is shown with a blue frame.

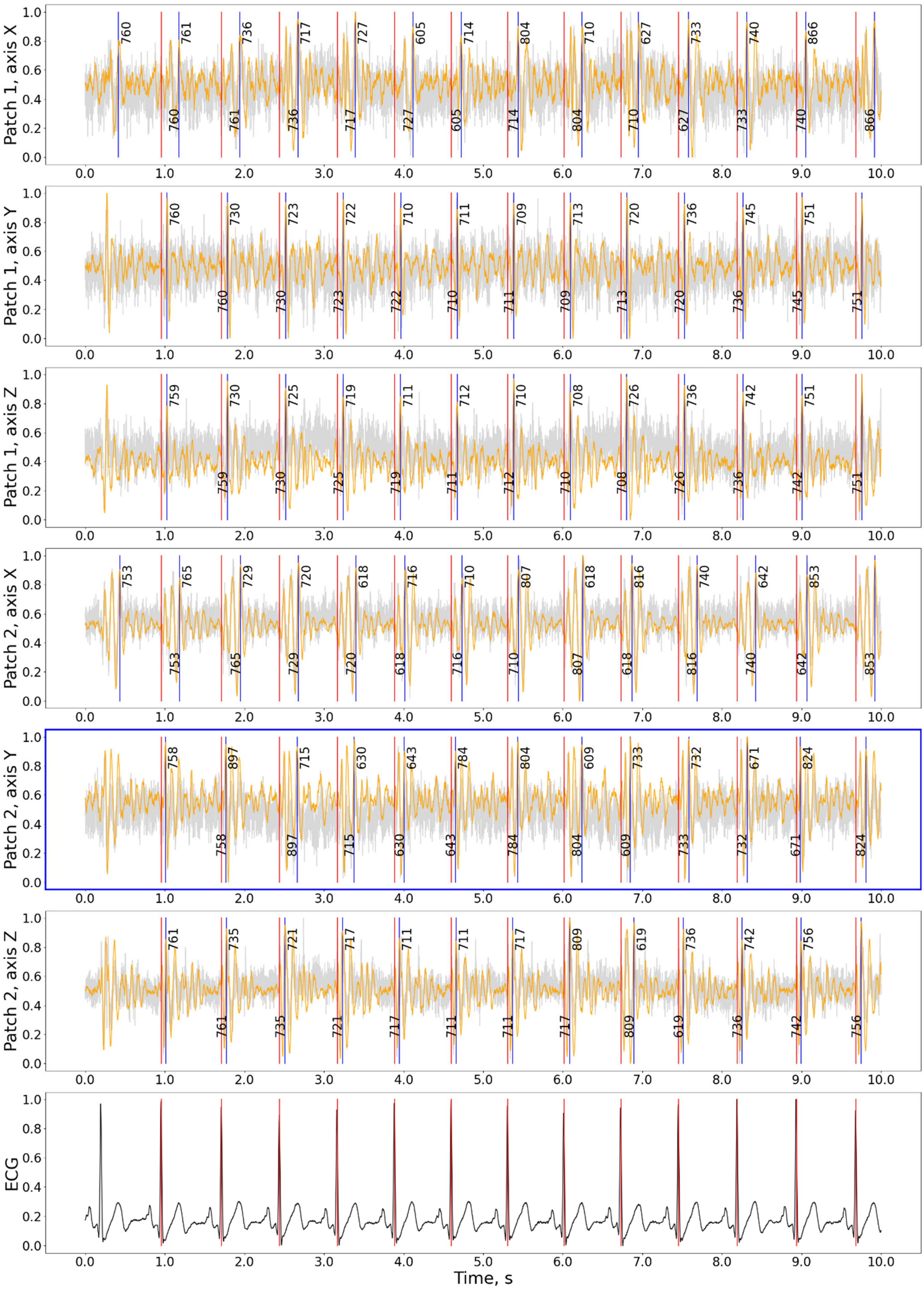
Patch 1, axis X
760 761 736 717 727 605 714 804 710 627 733 740 866
Patch 1, axis Y
760 730 723 722 710 711 709 713 720 736 745 751
Patch 1, axis Z
759 730 725 719 711 712 710 708 726 736 742 751
Patch 2, axis X
753 765 729 720 618 716 710 807 618 816 740 642 853
Patch 2, axis Y
758 897 715 630 643 784 804 609 733 732 671 824
Patch 2, axis Z
761 735 721 717 711 711 717 809 619 736 742 756
ECG
Time, s

Figure S2. The detection results for the hamilton2002 preprocessing and nabian2018 peak detection combination on the experimental data. Subject 2, relaxed. Gray curve – raw signal, orange curve – signal after being processed with hamilton2002 algorithm, black curve in the bottom plot – ECG signal, red vertical lines – timestamps of R-peaks detected on ECG, blue vertical lines – J-peaks detected on SCG, the numbers around blue vertical lines show the distance from the previous and to the next peak in ms. The patch with the best precision is shown with a blue frame.

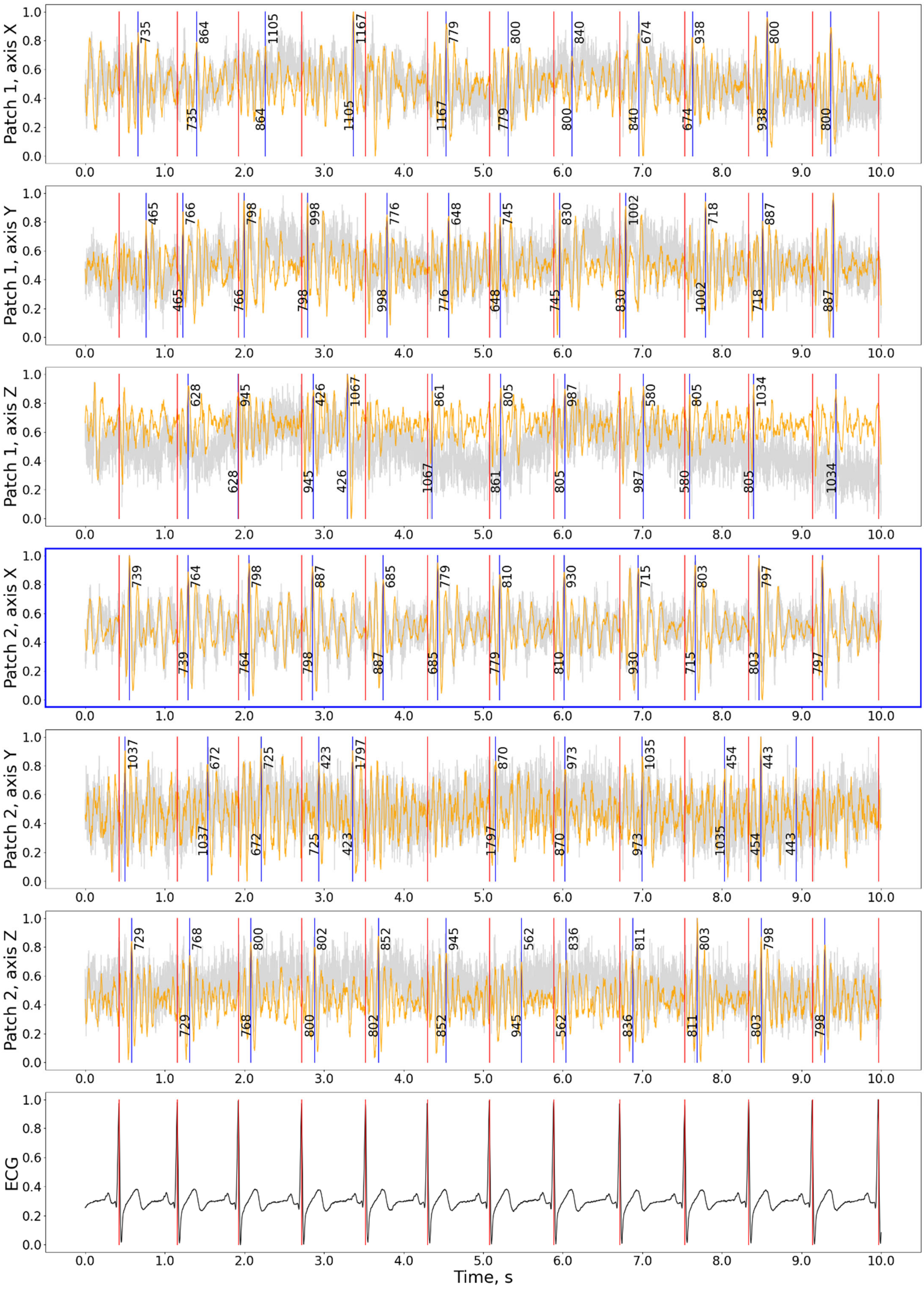

Patch 1, axis X
Patch 1, axis Y
Patch 1, axis Z
Patch 2, axis X
Patch 2, axis Y
Patch 2, axis Z
ECG
Time, s

Figure S3. The detection results for the hamilton2002 preprocessing and nabian2018 peak detection combination on the experimental data. Subject 3, relaxed. Gray curve – raw signal, orange curve – signal after being processed with hamilton2002 algorithm, black curve in the bottom plot – ECG signal, red vertical lines – timestamps of R-peaks detected on ECG, blue vertical lines – J-peaks detected on SCG, the numbers around blue vertical lines show the distance from the previous and to the next peak in ms. The patch with the best precision is shown with a blue frame.

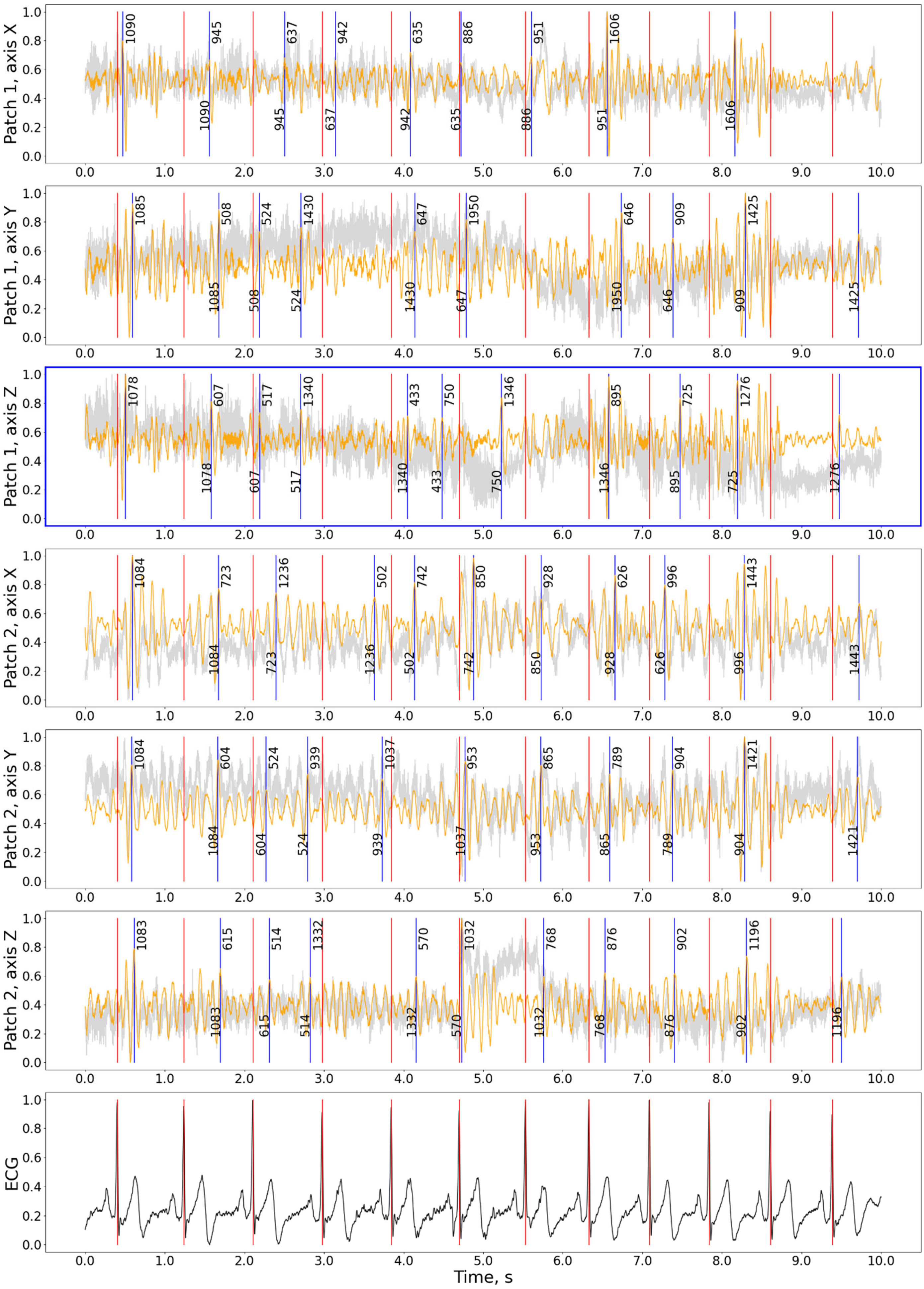
Patch 1, axis X
1090 945 637 942 635 886 951 1606
Patch 1, axis Y
1085 508 524 1430 647 1950 646 909 1425
Patch 1, axis Z
1078 607 517 1340 433 750 1346 895 725 1276
Patch 2, axis X
1084 723 1236 502 742 850 928 626 996 1443
Patch 2, axis Y
1084 604 524 939 1037 953 865 789 904 1421
Patch 2, axis Z
1083 615 514 1332 570 1032 768 876 902 1196
ECG
0.0 0.2 0.4 0.6 0.8 1.0
0.0 1.0 2.0 3.0 4.0 5.0 6.0 7.0 8.0 9.0 10.0
Time, s

Figure S4. The detection results for the hamilton2002 preprocessing and nabian2018 peak detection combination on the experimental data. Subject 1, interference. Gray curve – raw signal, orange curve – signal after being processed with hamilton2002 algorithm, black curve in the bottom plot – ECG signal, red vertical lines – timestamps of R-peaks detected on ECG, blue vertical lines – J-peaks detected on SCG, the numbers around blue vertical lines show the distance from the previous and to the next peak in ms. The patch with the best precision is shown with a blue frame.

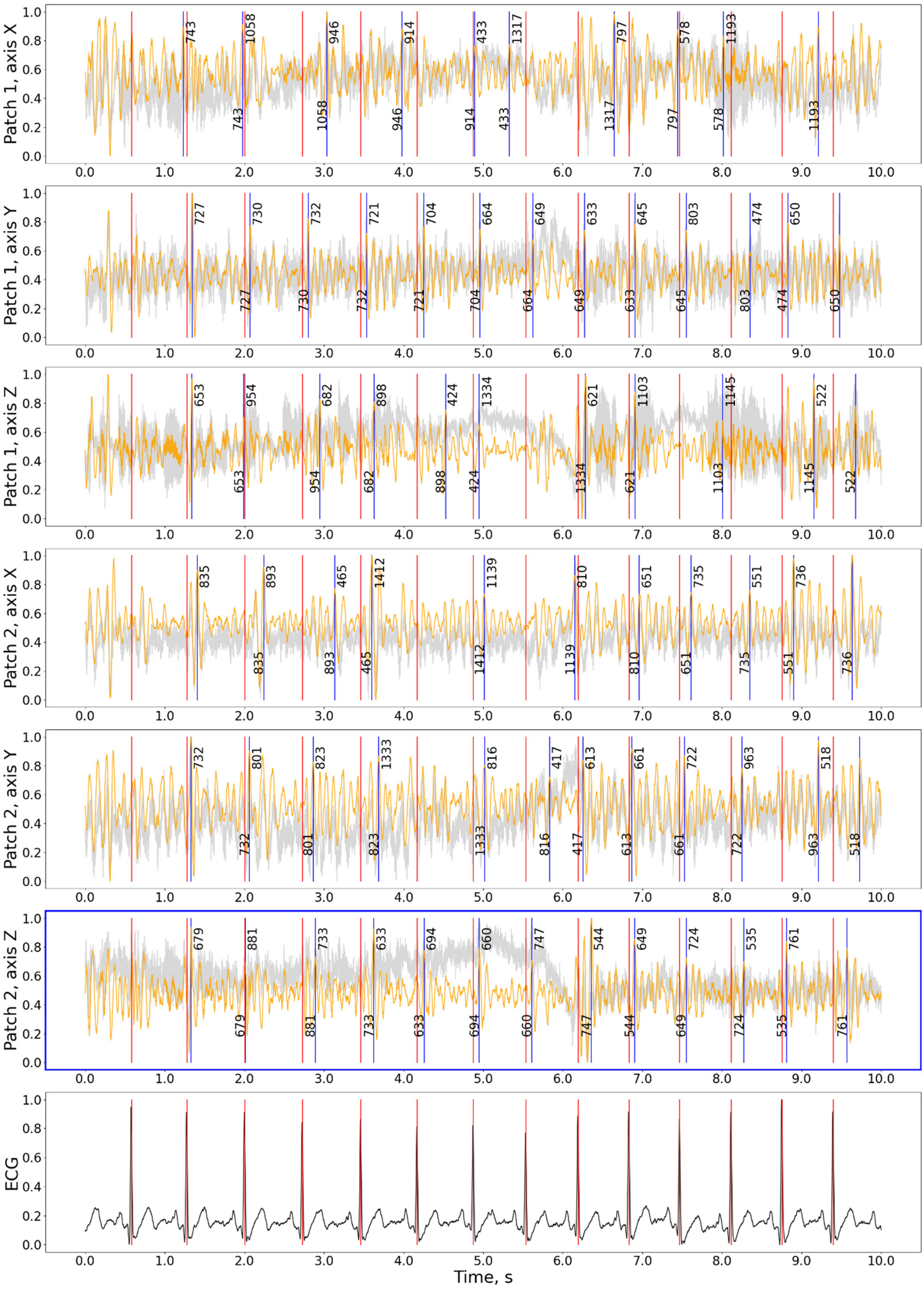
Patch 1, axis X
743 1058 946 914 433 1317 797 578 1193
Patch 1, axis Y
727 730 732 721 704 664 649 633 645 803 474 650
Patch 1, axis Z
653 954 682 898 424 1334 621 1103 1145 522
Patch 2, axis X
835 893 465 1412 1139 810 651 735 551 736
Patch 2, axis Y
732 801 823 1333 816 417 613 661 722 963 518
Patch 2, axis Z
679 881 733 633 694 660 747 544 649 724 535 761
ECG
0.0 0.2 0.4 0.6 0.8 1.0
0.0 1.0 2.0 3.0 4.0 5.0 6.0 7.0 8.0 9.0 10.0
Time, s

Figure S5. The detection results for the hamilton2002 preprocessing and nabian2018 peak detection combination on the experimental data. Subject 2, interference. Gray curve – raw signal, orange curve – signal after being processed with hamilton2002 algorithm, black curve in the bottom plot – ECG signal, red vertical lines – timestamps of R-peaks detected on ECG, blue vertical lines – J-peaks detected on SCG, the numbers around blue vertical lines show the distance from the previous and to the next peak in ms. The patch with the best precision is shown with a blue frame.

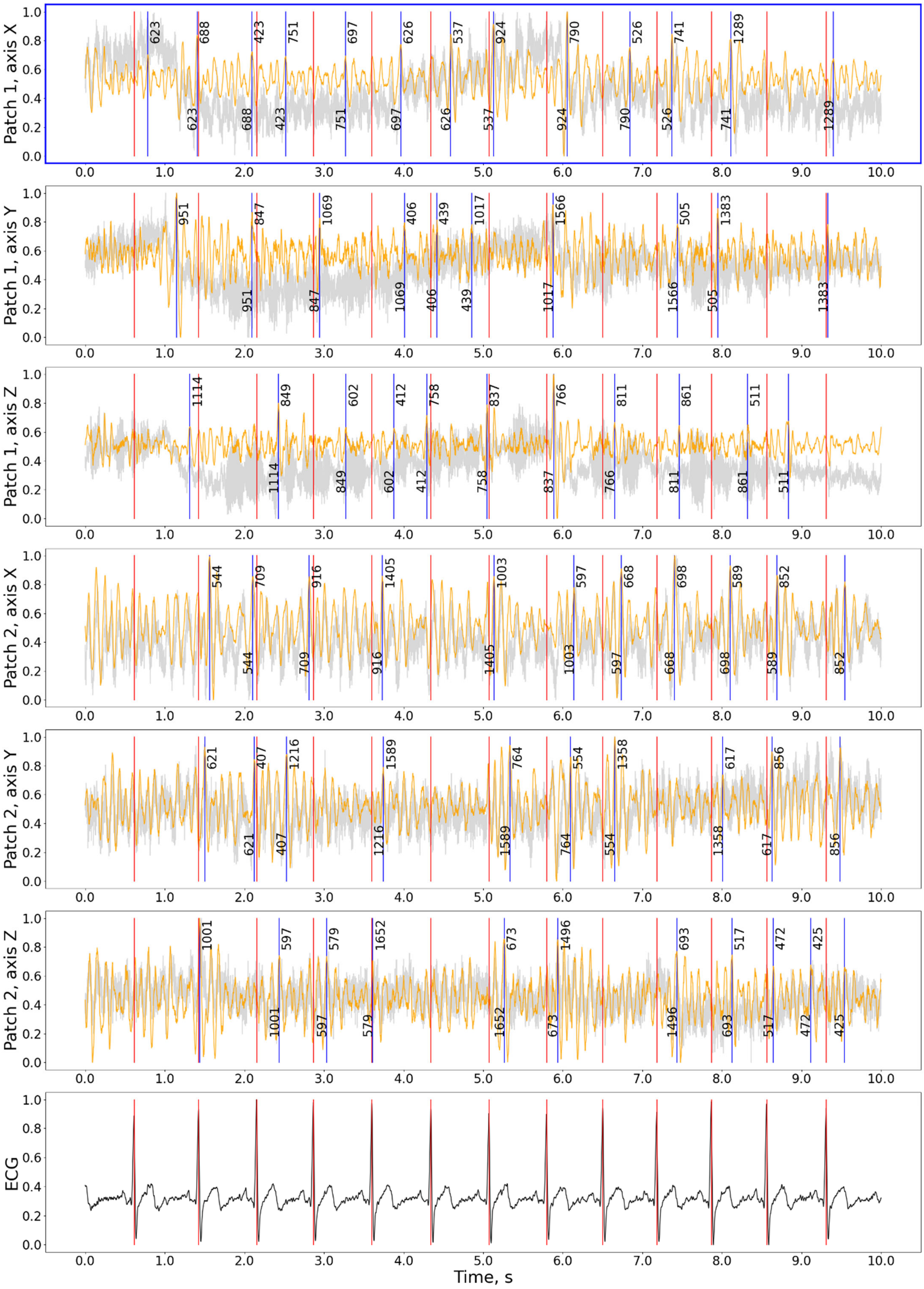
Patch 1, axis X
Patch 1, axis Y
Patch 1, axis Z
Patch 2, axis X
Patch 2, axis Y
Patch 2, axis Z
ECG
0.0
0.2
0.4
0.6
0.8
1.0
0.0
1.0
2.0
3.0
4.0
5.0
6.0
7.0
8.0
9.0
10.0
Time, s

Figure S6. The detection results for the hamilton2002 preprocessing and nabian2018 peak detection combination on the experimental data. Subject 3, interference. Gray curve – raw signal, orange curve – signal after being processed with hamilton2002 algorithm, black curve in the bottom plot – ECG signal, red vertical lines – timestamps of R-peaks detected on ECG, blue vertical lines – J-peaks detected on SCG, the numbers around blue vertical lines show the distance from the previous and to the next peak in ms. The patch with the best precision is shown with a blue frame.
